# Supplementary material for: Protein Conformational Dynamics Underlie Selective Recognition of Thermophilic over Mesophilic Enzyme I by a Substrate Analogue
Source: Biomolecules. 2023 Jan 12;13(1):160. doi: 10.3390/biom13010160 (PMC9856155; doi:10.3390/biom13010160)
Supplement: Supplementary file 1 [file biomolecules-13-00160-s001.zip › biomolecules-2116811-supplementary.pdf]

SUPPLEMENTARY MATERIAL

Protein conformational dynamics underlay selective recognition of thermophilic over mesophilic Enzyme I by a substrate analogue

Aayushi Singh[1], Daniel Burns[2], Sergey L. Sedinkin[1], Brett VanVeller[1], Davit A. Potoyan[1,2,*] and Vincenzo Venditti[1,2,*]

[1] *Department of Chemistry, Iowa State University, Ames, Iowa 50011, USA.*

[2] *Roy J. Carver Department of Biochemistry, Biophysics and Molecular Biology, Iowa State University, Ames, Iowa 50011, USA.*

*Address correspondence to:

Vincenzo Venditti, Department of Chemistry, Iowa State University, Hach Hall, 2438 Pammel Drive, Ames, IA 50011, USA. Email: venditti@iastate.edu; Tel: 515-294-1044; Fax: 515-294-7550; ORCID 0000-0001-8734-0400.

Davit A. Potoyan, Department of Chemistry, Iowa State University, Gilman Hall, 2415 Osborn Drive, Ames, IA 50011, USA. Email: potoyan@iastate.edu; Tel: 515-294-9971; Fax: 515-294-7550.

X: active site **X**: conserved residues —EIN —EIC - - - active site loops *: mutations

```
eEI  1    MISGILASPGIAFGKALLLKEDEIVIDRKKISADQVDQEVERFLSGRAKASAQLETIKTK  60
tEI  1    MLKGVAASPGIAIGKAFLYTKEKVTINVEKIEESKVEEEIAKFRKALEVTQEEIEKIKEK  60

eEI  61   AGETFGEEKEAIFEGHIMLLEDEELEQEIIALIKDKHMTADAAAHEVIEGQASALEELDD  120
tEI  61   ALKEFGKEKAEIFEAHLMLASDPELIEGVENMIKTELVTADNAVNKVIEQNASVMESLND  120

eEI  121  EYLKERAADVRDIGKRLLRNILGLKIIDLSAIQDEVILVAADLTPSETAQLNLKKVLGFI  180
tEI  121  EYLKERAVDLRDVGNRIIENLLGVKSVNLSDLEEEVVVIARDLTPSDTATMKKEMVLGFA  180

eEI  181  TDAGGRTSHTSIMARSLELPAIVGTGSVTSQVKNDDYLILDAVNNQVYVNPTNEVIDKMR  240
tEI  181  TDVGGRTSHTAIMARSLEIPAVVGLGNVTSQVKAGDLVIVDGLEGIVIVNPDEKTVEDYK  240

                                               **
eEI  241  AVQEQVASEKAELAKLKDLPAITLDGHQVEVCANIGTVRDVEGAERNGAEGVGLYRTEFL  300
tEI  241  SKKESYEKKVEGLKQLKDLPAETPDGKKVMLAANIGTPKDVASALANGAEGVGLFRTEFL  300
                                                              β2α2 loop
          *   **  *                        *          ***   *     *
eEI  301  FMDRDALPTEEEQFAAYKAVAEACGSQAVIVRTMDIGGDKELPYMNFPKEENPFLGWRAI  360
tEI  301  YMDRNSLPSEEEQFEAYKEVVEKMGGRPVTIRTLDIGGDKELPYLDMPKEMNPFLGYRAI  360
          (res. 296-309)                    β3α3 loop (res. 332-360)

eEI  361  RIAMDRKEILRDQLRAILRASAFGKLRIMFPMIISVEEVRALRKEIEIYKQELRDEGKAF  420
tEI  361  RLCLDRPDIFKTQLRAILRASAYGNVQIMYPMISSVEEVRKANSILEEVKAELDREGVKY  420

                                                         * ******   **
eEI  421  DESIEIGVMVETPAAATIARHLAKEVDFFSIGTNDLTQYTLAVDRGNDMISHLYQPMSPS  480
tEI  421  DKEIKVGIMVEIPSAAVTADILAKEVDFFSIGTNDLTQYTLAVDRMNEHVKEYYQPFHPA  480
                                        β6α6 loop (res. 454-477)

eEI  481  VLNLIKQVIDASHAEGKWTGMCGELAGDERATLLLLGMGLDEFSMSAISIPRIKKIIRNT  540
tEI  481  ILRLVKMVIDAAHKEGKFAAMCGEMAGDPLAAVILLGLGLDEFSMSATSIPEIKNIIRNV  540

eEI  541  NFEDAKVLAEQALAQPTTDELMTLVNKFIEEKTIC  575
tEI  541  EYEKAKEIAEKALNMSEAREIEKMMKDVI--KDIG  573
```

Figure S1. Sequence alignment of eEI and tEI. The amino acid sequences of eEI and tEI were aligned in BLAST. Active site residues are colored red. Conserved residues are colored green. The EIN and EIC domains are indicated with a blue and red line underneath the amino acid sequence, respectively. The location of the active site loops is shown with dashed black lines underneath the amino acid sequence. Positions of the 21 single-point mutations performed to hybridize the EIC domain are indicated by asterisks over the amino acid sequence. Note that two mutations (at positions 278 and 279) lay outside of the β2α2, β3α3, and β6α6 loops. These residues were mutated because facing the β2α2 loop in the 3D structures of EIC (see reference 16 in the main text).

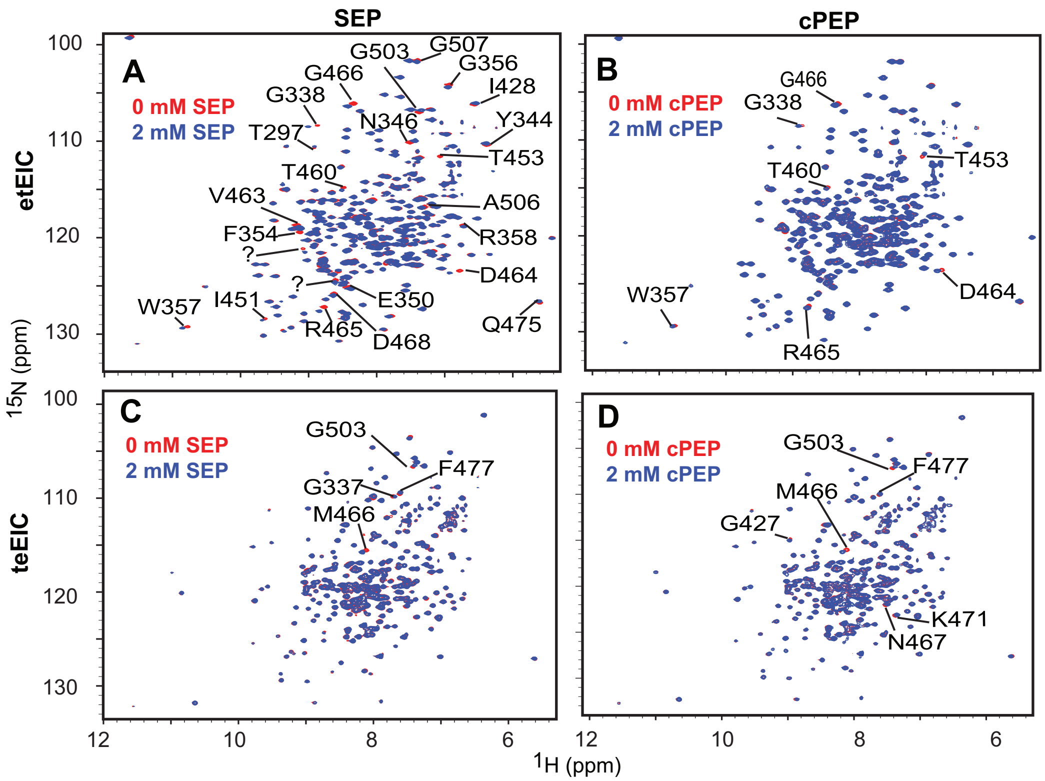

Figure S2. 800 MHz ^{1}H-^{15}N TROSY spectra of etEIC and teEIC measured in the absence (red) and in the presence (blue) of 2 mM SEP or cPEP. Panels **(A)**, **(B)**, **(C)**, and **(D)** display the data measured for the etEIC-SEP, etEIC-cPEP, teEIC-SEP, and teEIC-cPEP systems, respectively. Peaks that shift upon addition of ligand are assigned in the spectra. "?" indicates peaks with unknown assignment.

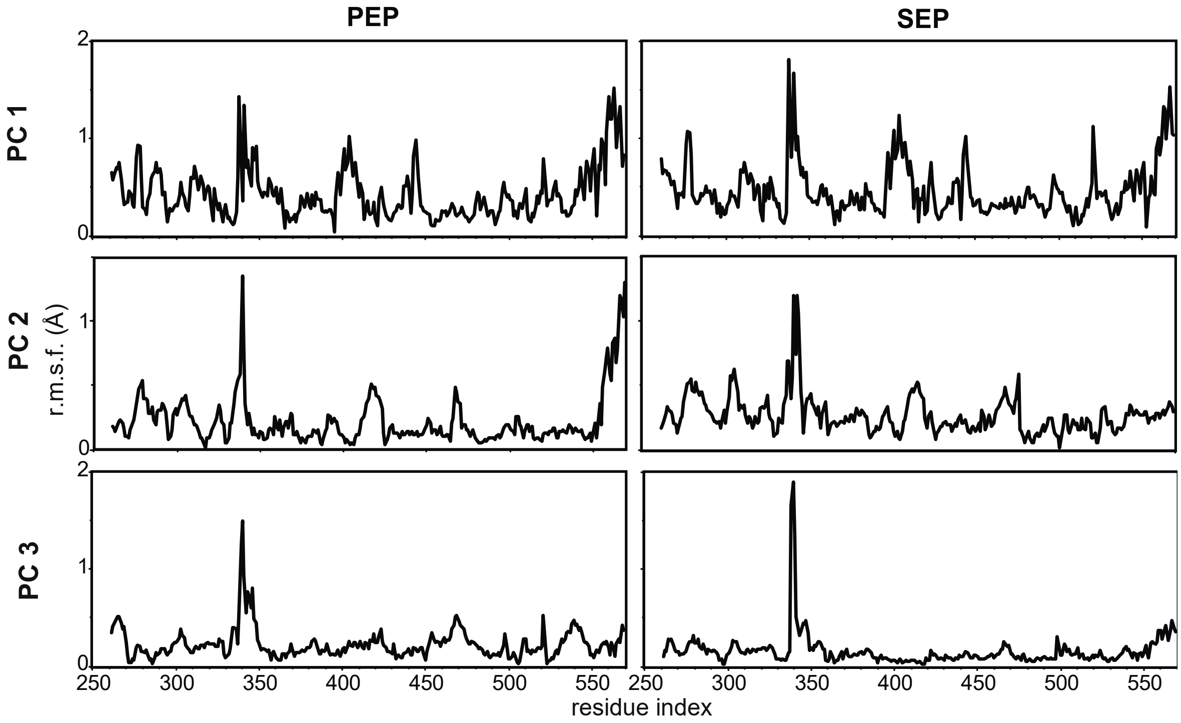

Figure S3. Residue-specific r.m.s.f. values (relative to the average structure) in PC 1 (top), PC 2 (middle), and PC 3 (bottom) calculated over the concatenated trajectory of the EIC-PEP (left) and EIC-SEP (right) complexes are plotted versus the residue index to emphasize the specific contribution of different EIC regions to each PC.
